# The influence of student factors on students’ achievement in the Trends in International Mathematics and Science Study in Abu Dhabi Emirate Schools

**DOI:** 10.3389/fpsyg.2023.1168032

**Published:** 2023-05-10

**Authors:** Yousef Wardat, Shashidhar Belbase, Hassan Tairab, Rachel Alison Takriti, Maria Efstratopoulou, Hamza Dodeen

**Affiliations:** ^1^Higher Colleges of Technology, Al Ain, United Arab Emirates; ^2^Department of Curriculum and Instruction, College of Education, United Arab Emirates University, Al Ain, United Arab Emirates; ^3^Department of Special Education, College of Education, United Arab Emirates University, Al Ain, United Arab Emirates; ^4^Department of Cognitive Sciences, College of Humanities and Social Sciences, United Arab Emirates University, Al Ain, United Arab Emirates

**Keywords:** student factors, student achievement, TIMSS study, regression, performance in mathematics

## Abstract

**Introduction:**

This study aimed to investigate student factors affecting performance in mathematics in Abu Dhabi schools in the United Arab Emirates.

**Method:**

We used the secondary data from the Trends in International Mathematics and Science Study (TIMSS) 2015, which included 4,838 eighth-grade students from 156 schools in Abu Dhabi.

**Result:**

The data from the student questionnaire in TIMSS 2015 were subjected to principal component analysis (PCA). The 39 questions were reduced to five factors generated from the student questionnaire, including Safety and Behavior, Classroom Mathematics, Environment, Student Attitudes toward Mathematics, and Technology and Resources. The effects of these factors on students’ achievement were examined using multiple regression analysis.

**Discussion:**

All of these factors had a significant impact on student achievement in the 2015 TIMSS. The pedagogical and policy implications of the findings have been discussed.

## 1. Introduction

The Trends in International Mathematics and Science Study (TIMSS) has been a major international assessment to compare students’ achievement across economies. This study also provided a robust database for researchers to assess various factors related to students’ achievements in mathematics and science. TIMSS was first administered in 1995 and has been repeated every 4 years since then. According to the findings from TIMSS 2015, students from Abu Dhabi schools performed below the international average in mathematics (Fulmer et al., 2018). Fourth-grade students from Abu Dhabi ranked 36th, and eighth-grade students ranked 24th in the TIMSS 2015 mathematics assessment. The findings revealed the need to evaluate the predictors of mathematical achievement. In TIMSS 2015, we explored the role of school factors in predicting the mathematical achievement of Abu Dhabi eighth-grade pupils, taking these aspects into account. The influence of school elements can be predicted by identifying major and dependable factors. The study’s findings are expected to help schools achieve the desired beneficial outcomes in mathematics (Al Shannag et al., 2013). In this context, the current study aimed to identify the student factors affecting mathematics achievement at TIMSS 2015 in Abu Dhabi. The research question for the study was, What are the student factors that may predict student mathematics achievement in TIMSS 2015 in Abu Dhabi schools? The novelty of this study is that it reshuffled the items in the student questionnaire in TIMSS 2015 for Abu Dhabi, United Arab Emirates, to reassess the student factors into a small number of factors that influenced eighth-grade students’ achievement in mathematics (Achtziger and Gollwitzer, 2018; Goodall, 2018). This re-alignment is based on items’ correlations (factor loading coefficients) to each other, which is more robust than the original item grouping conducted manually. The findings of the study within a few factors would be more manageable than the list of many factors that might have influenced students’ achievement in mathematics (Wardat et al., 2022b).

### 1.1. Student factors in mathematics achievements

The influence of student variables on performance in various mathematical assessments has been evaluated by considering the findings of different studies. Lamb and Fullarton (2002) conducted a cross-national study of student, classroom, and school factors influencing mathematics achievement in the United States of America (USA) and Australia. The study’s data depended on the TIMSS 1995 to determine the impact of students’ related mathematics achievement variables. To this end, the study employed hierarchical linear modeling (HLM) by Bryk and Raudenbush (1988).

The study adopted two analyses to examine the differences in academic performance between American and Australian students. To illustrate, the first set tackled the following students’ variables: gender, language background, family size, socioeconomic status, families of single parents, and parents’ place of birth. Moreover, the study accounted for the time students spent on homework, their attitude toward mathematics, and its importance. The findings revealed that student characteristics accounted for only 4.7% of the total variance, whereas this percentage for Australia was 7.4%. While readjusting for the mediating variables associated with classroom and school, the percentage contribution of student variables changed to 12 and 19.3% for the US and Australia, respectively.

Yalcin et al. (2017) studied the impact of student characteristics on math achievement in the TIMSS 2011 for grades four and eight in Turkey. The study applied a HLM analysis. Most importantly, the ANOVA model was used for both fourth- and eighth-grade students to understand the variations in school performance in mathematics according to TIMSS 2011. The findings revealed that the more students were forced to study, the less likely they were to achieve high mathematics performance. On the contrary, the more the students were willing to study, the greater their mathematics achievement (Mullis and Martin, 2012).

Many researchers have attempted to study the underlying factors related to student learning, especially in mathematics, physics, and other allied science subjects, in the past two decades. These previously undertaken studies have found that there is a consistent relationship between some of the associated background measurements that comprise the size of the family, ethnicity, and socio-economic status, as well as the learning capability and interests of the students, which are seldom associated with the form of determinants toward student outcomes (Acharya, 2017).

Fung et al. (2018) conducted research that uncovered the primary and interactive effects of research on student engagement and math achievement. The PISA research only included 15-year-old pupils from 11,767 secondary schools in 34 participating nations (2018). The study applied a HLM analysis, an independent *t*-test, and a three-level fixed-effect HLM with total maximum likelihood estimation. In addition, independent sample *t*-tests were next employed to examine whether students benefited from having higher levels of engagement in two different components simultaneously (Mullis et al., 2016; National Center for Education Statistics, 2020). For example, they studied students with high and low motivation in mathematics. The findings revealed that the more students engaged in learning mathematics with greater motivation, the higher their mathematics achievement (Fung et al., 2018). Student achievement may be significantly impacted by confidence levels due to their motivation. It was revealed in a research study that students’ confidence in mathematics had a considerable impact on their achievements in 2011, not in 2007. Students’ valuation of mathematics variables did not significantly correlate with students’ mathematics achievement in either year (Lee and Stankov, 2018).

As shown above, students’ academic performance is influenced by several personal and environmental factors. As Farooq et al. (2011) noted, these factors include parental background, peer influence, and learning skills. A supportive parental background can positively impact a student’s performance by fostering a love for learning. Students from stable homes that provide a positive and stress-free environment tend to perform better than those from less stable backgrounds. Peer influence also plays a significant role, with positive friends serving as a source of motivation, while negative ones can lead to a decline in academic performance (Harris and Sass, 2011; Herges et al., 2017). Finally, learning skills, such as comprehending and practicing concepts, are critical to a student’s academic performance.

## 2. Materials and methods

This study employs a mixed-methods approach to investigate the factors that affect student achievement in mathematics and science subjects. The research utilized a specific data source and sample, and the instruments and data collection methods were carefully selected to ensure validity and reliability.

### 2.1. TIMSS 2015 data

Trends in International Mathematics and Science Study 2015 data on eighth-grade students’ math proficiency and their reports in the questionnaires were used in this study. Science and mathematics have been evaluated by TIMSS in 1995, 1999, 2003, 2007, 2011, and 2019 (Martin et al., 2016). A wide range of student background data is gathered by TIMSS. In this study, TIMSS 2015 was conducted in 63 countries worldwide, with a total of 425,000 students participating from all of these countries (TIMSS, 2015). Additionally, in Abu Dhabi, 257 public schools are managed by the Abu Dhabi Education Council (ADEC) in Abu Dhabi. The Abu Dhabi Emirate also has 188 more private schools in operation. There were 127,770 students in public schools and 223,803 students in private schools in Abu Dhabi in 2015.^1^ The student body at public schools comprises 77% Emirati citizens and 23% foreigners. A total of 76% of the students in private schools are foreigners, while 24% are Emirati citizens (see text footnote 1).

### 2.2. Questionnaires

The data from TIMSS 2015 included a student questionnaire and student achievement in math with five plausible values. The student questionnaire contained 90 forced-choice questions that covered student background factors, home possessions, attitudes toward mathematics, learning mathematics, and perceptions about the school climate. We excluded all science-related data and used only student variables and mathematics achievement data in our analysis of TIMSS 2015 for Abu Dhabi schools (Table 1).

**TABLE 1:** Students questionnaire sections (each section has multiple items).

Categories	Number of questions
About you	1–14
Your school	15–16
Mathematics in school	17–20
Homework	25–26

### 2.3. Study sample

The sample for this study consisted of all the eighth-grade students who participated in TIMSS 2015 in Abu Dhabi Emirate Schools. A total of 156 schools took part in TIMSS 2015 for eighth-grade math in Abu Dhabi. The number of students was 4,838, with an average age of 13.9 years, who participated in TIMSS 2015 in Abu Dhabi (Martin et al., 2012). Of the 1,838 students who participated in TIMSS 2015 in Abu Dhabi, 2,666 were males and 2,172 were females. We integrated the eighth-grade student questionnaire and achievement in mathematics into TIMSS 2015.

### 2.4. TIMSS 2015 data

The data collection procedure for TIMSS 2015 has been described by Johansson (2016) in terms of the 60 participating countries that were spread out throughout the southern and northern hemispheres. For the southern hemisphere, the academic year normally finishes in November or December, and the TIMSS 2015 was administered in October or November. The evaluation was completed in the northern hemisphere in April, May, or June 2015. The survey and assessment operations methods were established and standardized as per the standards established by the IEA for the TIMSS 2015 (Johansson, 2016).

Data from the TIMSS math test for eighth graders can be divided into two primary categories: content domains and cognitive domains. The content areas covered the real number system, algebra, geometry, statistics, and probabilities. The cognitive areas covered knowledge, application, and thinking (Allison, 2001). They are categorized and displayed in tables to compare and analyze how these two regions are related (Allison, 2001).

Factors that correlated to the students’ performance in TIMSS, as described by Kromrey and Rendina-Gobioff (2006), included the interaction of students with peers, the interaction between students and teachers, the scoring pattern, and the reliability of significant benefits over students. All these variables were extracted from the students’ questionnaire, which, in turn, was then described and analyzed. For students, the factors include kids’ motivation to succeed academically, their capacity to do so, their esteem for their peers who achieve academic success, and the clarity of the educational goals of the school (Kromrey and Rendina-Gobioff, 2006).

### 2.5. Analysis

To begin, we used the principal component analysis (PCA) to condense 90 items from the TIMSS 2015 student survey into a few categorical variables that were related to mathematics achievement. The results of each categorical variable were then examined item-wise to see if they differed significantly from the hypothesized mean scores of student perceptions using one-sample *t*-tests. The student factors were then examined to see if they had a significant effect on eighth-grade students’ mathematics achievement in TIMSS 2015 using multiple linear regression analysis.

## 3. Results

This study used quantitative data analysis, including factor analysis, descriptive analysis, a one-sample *t*-test, and multiple regression.

### 3.1. Principal component analysis

We utilized a PCA to reduce the 90 items from the eighth-grade student questionnaire into five dominant factors related to students in TIMSS 2015 for Abu Dhabi. The overall Kaiser–Meyer–Olkin (KMO) measure was 0.944, classified as marvelous (0.9 ≤ KMO) (Kaiser, 1960). The test of sphericity was significant at a *p* < 0.05 level of significance (Table 2).

**TABLE 2:** Kaiser–Meyer–Olkin and Bartlett’s test.

KMO and Bartlett’s test		
Kaiser–Meyer–Olkin measure of sampling adequacy		0.944
Bartlett’s test of sphericity	Approximately Chi-square	38,549.689
	df	4,005
	Significant	0.000

**FIGURE 1:**
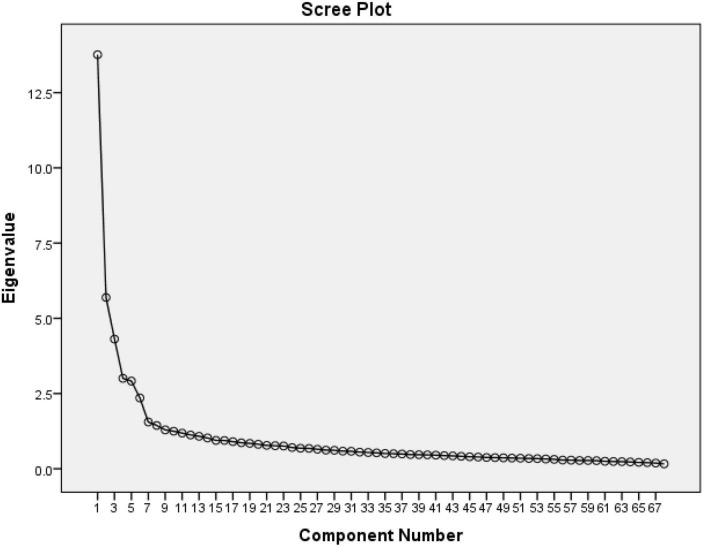
Plot of eigenvalues from exploratory factor analysis of the students’ questionnaire variables.

We derived five principal factors with eigenvalues greater than one (Figure 1). Although there were 19 potential factors with this criterion, the rest had a low-reliability coefficient. These five components accounted for 15.76, 5.89, 4.90, 3.79, and 3.25% of the total variances of the dependent variables, respectively. The scree plot also confirmed five potential components (Cattell, 1966). In addition, a five-component solution met the interpretability criterion. Therefore, we retained five components as the dominant student factors that might have affected their achievement in mathematics in TIMSS 2015 in Abu Dhabi schools (Table 3).

**TABLE 3:** Results of principal component analysis of student factors in TIMSS 2015 for Abu Dhabi schools.

	Initial eigenvalues			Extraction sums of squared loadings			Rotation sums of squared loadings		
**Component**	**Total**	**% of variance**	**Cumulative %**	**Total**	**% of variance**	**Cumulative %**	**Total**	**% of variance**	**Cumulative %**
1	14.184	15.760	15.760	14.184	15.760	15.760	12.919	14.355	14.355
2	5.297	5.886	21.646	5.297	5.886	21.646	5.086	5.651	20.006
3	4.407	4.897	26.543	4.407	4.897	26.543	4.568	5.076	25.082
4	3.413	3.792	30.335	3.413	3.792	30.335	4.487	4.985	30.067
5	2.928	3.253	33.588	2.928	3.253	33.588	3.169	3.522	33.588
6	2.725	3.028	36.616						
7	2.045	2.272	38.888						
8	1.816	2.018	40.906						

The five factors were selected based on four criteria: First, an eigenvalue greater than one yielded 19 components. Second, retaining the first five components, we used a cumulative percentage variance greater than 50%. Third, we used the scree plot with sharp elbow points denoting the potential number of components to retain (five in this case). Fourth, we used the internal consistency reliability with Cronbach’s alpha to retain the components with the higher internal consistency of 0.957, 0.854, 0.818, 0.842, and 0.601, respectively (Table 4). The fifth criterion we used to confirm the number of components was the number of items loaded in each component that would make the potential interpretation meaningful (Straub et al., 2004).

**TABLE 4:** Student’s questionnaire factor analysis and reliability statistic.

No.	Item code	Item	Factor loading	Cronbach’s alpha	Students factors
1.	BSBM18E	MATH\AGREE\TEACHER CLEAR ANSWERS	0.718	0.957	Factor 1: Classroom Mathematics
2.	BSBM18D	MATH\AGREE\INTERESTING THINGS TO DO	0.712		
3.	BSBM18F	MATH\AGREE\TEACHER EXPLAINS GOOD	0.706		
4.	BSBM18G	MATH\AGREE\TEACHER SHOWS LEARNED	0.688		
5.	BSBM18B	MATH\AGREE\TEACHER IS EASY TO UNDERSTAND	0.685		
6.	BSBM18I	MATH\AGREE\TELLS HOW TO DO BETTER	0.681		
7.	BSBM18C	MATH\AGREE\INTERESTED IN WHAT TCHR SAYS	0.677		
8.	BSBM18H	MATH\AGREE\DIFFERENT THINGS TO HELP	0.671		
9.	BSBM18J	MATH\AGREE\TEACHER LISTENS	0.666		
10.	BSBM20A	MATH\AGREE\MATHEMATICS WILL HELP ME	0.665		
11.	BSBM17H	MATH\AGREE\LOOK FORWARD TO MATH CLASS	0.664		
12.	BSBM20F	MATH\AGREE\GET AHEAD IN THE WORLD	0.663		
13.	BSBM20B	MATH\AGREE\NEED MAT TO LEARN OTHER THINGS	0.652		
14.	BSBM17D	MATH\AGREE\LEARN INTERESTING THINGS	0.652		
15.	BSBM17E	MATH\AGREE\LIKE MATHEMATICS	0.646		
16.	BSBM17I	MATH\AGREE\FAVORITE SUBJECT	0.635		
17.	BSBM20G	MATH\AGREE\MORE JOB OPPORTUNITIES	0.635		
18.	BSBM19D	MATH\AGREE\LEARN QUICKLY IN MATHEMATICS	0.631		
19.	BSBM20I	MATH\AGREE\IMPORTANT TO DO WELL IN MATH	0.624		
20.	BSBM17G	MATH\AGREE\LIKE MATH PROBLEMS	0.623		
21.	BSBM20C	MATH\AGREE\NEED MATH TO GET INTO <UNI>	0.618		
22.	BSBM19G	MATH\AGREE\I AM GOOD AT MATHEMATICS	0.616		
23.	BSBM17F	MATH\AGREE\LIKE NUMBERS	0.611		
24.	BSBM17A	MATH\AGREE\ENJOY LEARNING MATHEMATICS	0.609		
25.	BSBM20E	MATH\AGREE\JOB INVOLVING MATHEMATICS	0.609		
26.	BSBM20D	MATH\AGREE\NEED MAT TO GET THE JOB I WANT	0.607		
27.	BSBM19F	MATH\AGREE\GOOD AT WORKING OUT PROBLEMS	0.557		
28.	BSBM18A	MATH\AGREE\TEACHER EXPECTS TO DO	0.555		
29.	BSBM20H	MATH\AGREE\PARENTS THINK MATHS IMPORTANT	0.535		
30.	BSBM19A	MATH\AGREE\USUALLY DO WELL IN MATH	0.526		
1.	BSBG16I	GEN\HOW OFTEN\THREATENED	0.738	0.854	Factor 2: Safety and Behavior
2.	BSBG16G	GEN\HOW OFTEN\EMBARRASSING INFO	0.727		
3.	BSBG16F	GEN\HOW OFTEN\FORCE TO DO STH	0.701		
4.	BSBG16E	GEN\HOW OFTEN\HURT BY OTHERS	0.690		
5.	BSBG16H	GEN\HOW OFTEN\POSTED EMBARRASSING THINGS	0.683		
6.	BSBG16C	GEN\HOW OFTEN\SPREAD LIES ABOUT ME	0.653		
7.	BSBG16B	GEN\HOW OFTEN\LEFT OUT OF GAMES	0.632		
8.	BSBG16A	GEN\HOW OFTEN\MADE FUN OF	0.588		
9.	BSBG16D	GEN\HOW OFTEN\STOLE STH FROM ME	0.562		
10.	BSBM39AA	MATH\EXTRA LESSONS LAST 12 MONTH\MATHEMATICS	0.322		
1.	BSBM19HRSTUDENT	MATH\AGREE\MATHEMATICS IS HARDER FOR ME TO REVERSE	0.701	0.818	Factor 3: Student Attitudes toward Math
2.	BSBM19CRSTUDENT	MATH\AGREE\MATHEMATICS NOT MY STRENGTH REVERSE	0.693		
3.	BSBM19ERSTUDENT	MATH\AGREE\MAT MAKES NERVOUS REVERSE	0.634		
4.	BSBM19BRSTUDENT	MATH\AGREE\MATHEMATICS IS MORE DIFFICULT TO REVERSE	0.633		
5.	BSBM17CRSTUDENT	MATH\AGREE\MATH IS BORING REVERSE	0.563		
6.	BSBM17BRSTUDENT	MATH\AGREE\WISH HAVE NOT TO STUDY MATH REVERSE	0.563		
7.	BSBM19IRSTUDENT	MATH\AGREE\MAT MAKES CONFUSED REVERSE	0.385		
1.	BSBG15G	GEN\AGREE\LEARN A LOT	0.715	0.842	Factor 4: Environment
2.	BSBG15F	GEN\AGREE\PROUD TO GO TO THIS SCHOOL	0.700		
3.	BSBG15B	GEN\AGREE\SAFE AT SCHOOL	0.693		
4.	BSBG15D	GEN\AGREE\LIKE TO SEE CLASSMATES	0.678		
5.	BSBG15C	GEN\AGREE\BELONG AT SCHOOL	0.668		
6.	BSBG15A	GEN\AGREE\BEING IN SCHOOL	0.612		
7.		GEN\AGREE\FAIR TEACHERS	0.596		
8.	BSBG03RSTUDENT	GEN\OFTEN SPEAK<LANG OF TEST> AT HOME REVERSE	0.401		
9.	BSBG15E	GEN\HOME POSSESS\STUDY DESK	0.324		
1.	BSBG06H	GEN\HOME POSSESS\<COUNTRY SPECIFIC>	0.502	0.601	Factor 5: Technology and Resources
2.	BSBG06G	GEN\HOME POSSESS\GAMING SYSTEM	0.472		
3.	BSBG06A	GEN\HOME POSSESS\COMPUTER TABLET OWN	0.461		
4.	BSBG06I	GEN\HOME POSSESS\<COUNTRY SPECIFIC>	0.442		
5.	BSBG13C	GEN\HOW OFTEN USE COMPUTER TABLET\OTHER	0.434		
6.	BSBG06J	GEN\HOME POSSESS\<COUNTRY SPECIFIC>	0.430		
7.	BSBG09B	GEN\FATHER BORN IN\<country>	0.402		
8.	BSBG06D	GEN\HOME POSSESS\OWN ROOM	0.370		
9.	BSBG09A	GEN\MOTHER BORN IN\<country>	0.363		
10.	BSBG06E	GEN\HOME POSSESS\INTERNET CONNECTION	0.358		
11.	BSBG06F	GEN\HOME POSSESS\OWN MOBILE PHONE	0.343		
12.	BSBG06K	GEN\HOME POSSESS\<COUNTRY SPECIFIC>	0.335		

All these criteria were met except the total cumulative variance explained by the five factors (or components), which, in this case, only explained approximately 41.0% of the total variance when a varimax orthogonal rotation was used that exhibited the structural alignment of items within these components (Thurstone, 1947). The first factor was named Classroom Mathematics. The second factor was named Safety and Behavior. The third factor was named Student Attitudes toward Mathematics. The fourth factor was named Environment. The fifth factor was named Technology and Resources.

(Reverse Item): This signifies that the numerical scoring scale is oriented differently. Therefore, for the aforementioned items, highly disagree would receive a score of 5, disagree would be 4, neutral would remain equal to 3, agree would become 2, and very agree would receive a score of 1.

Factor 1: Mathematics in School comprises 30 variables listed in Table 5 under the codes BSBM18E, BSBM18D, BSBM18F, etc. Factor 1’s Cronbach’s alpha (α) value is 0.957, which is higher than 0.9 and is regarded as “good and adequate” (Cho, 2010). It demonstrates the high level of internal consistency among the factor’s variables. As a result, factor 1 has great reliability among the variables. Each component variable for Factor 1: Mathematics in Schools had moderately high loadings (between 0.526 and 0.718), indicating that they reasonably well captured the underlying construct. For a scale with 30 variables, 15.76% of the variance in Factor 1 was explained by its constituent variables, which is a substantial amount. The component variables’ validity is further demonstrated by Cronbach’s alpha, which is 0.957. These variables functioned well together.

**TABLE 5:** One-sample statistics and *t*-test (test value = 2.5).

No.	Items	*N*	Mean	SD	Mean difference	*t*-Value	Significant (two-tailed)	Confident and not confident
1.	Factor 1: Mathematics in School	4,774	1.9552	0.62760	−0.54485	−59.984	.0.000	SN
2.	MATH\AGREE\TEACHER CLEAR ANSWERS	4,639	1.84	0.895	−0.664	−50.565	0.000	SN
3.	MATH\AGREE\INTERESTING THINGS TO DO	4,650	2.07	0.950	−0.428	−30.696	0.000	SN
4.	MATH\AGREE\TEACHER EXPLAINS GOOD	4,620	1.77	0.887	−0.726	−55.591	0.000	SN
5.	MATH\AGREE\TEACHER SHOWS LEARNED	4,629	1.91	0.898	−0.593	−44.896	0.000	SN
6.	MATH\AGREE\TEACHER IS EASY TO UNDERSTAND	4,659	1.93	0.893	−0.571	−43.631	0.000	SN
7.	MATH\AGREE\TELLS HOW TO DO BETTER	4,639	1.79	0.872	−0.706	−55.160	0.000	SN
8.	MATH\AGREE\INTERESTED IN WHAT TCHR SAYS	4,651	1.79	0.833	−0.706	−57.773	0.000	SN
9.	MATH\AGREE\DIFFERENT THINGS TO HELP	4,667	1.90	0.917	−0.604	−44.986	0.000	SN
10.	MATH\AGREE\TEACHER LISTENS	4,660	1.91	0.943	−0.587	−42.504	0.000	SN
11.	MATH\AGREE\MATHEMATICS WILL HELP ME	4,608	1.70	0.857	−0.798	−63.184	0.000	SN
12.	MATH\AGREE\LOOK FORWARD TO MATH CLASS	4,700	2.36	1.047	−0.137	−8.983	0.000	SN
13.	MATH\AGREE\GET AHEAD IN THE WORLD	4,569	1.76	0.856	−0.737	−58.237	0.000	SN
14.	MATH\AGREE\NEED MAT TO LEARN OTHER THINGS	4,600	1.89	0.876	−0.613	−47.514	0.000	SN
15.	MATH\AGREE\LEARN INTERESTING THINGS	4,654	2.04	0.976	−0.464	−32.442	0.000	SN
16.	MATH\AGREE\LIKE MATHEMATICS	4,662	2.13	1.057	−0.366	−23.617	0.000	SN
17.	MATH\AGREE\FAVORITE SUBJECT	4,727	2.37	1.131	−0.129	−7.811	0.000	SN
18.	MATH\AGREE\MORE JOB OPPORTUNITIES	4,568	1.71	0.847	−0.789	−62.981	0.000	SN
19.	MATH\AGREE\LEARN QUICKLY IN MATHEMATICS	4,595	2.05	0.923	−0.450	−33.073	0.000	SN
20.	MATH\AGREE\IMPORTANT TO DO WELL IN MATH	4,581	1.57	0.794	−0.932	−79.454	0.000	SN
21.	MATH\AGREE\LIKE MATH PROBLEMS	4,701	2.19	1.052	−0.307	−20.038	0.000	SN
22.	MATH\AGREE\NEED MATH TO GET INTO<UNI>	4,584	1.61	0.812	−0.889	−74.141	0.000	SN
23.	MATH\AGREE\I AM GOOD AT MATHEMATICS	4,575	2.06	0.934	−0.440	−31.852	0.000	SN
24.	MATH\AGREE\LIKE NUMBERS	4,695	2.26	1.009	−0.236	−16.047	0.000	SN
25.	MATH\AGREE\ENJOY LEARNING MATHEMATICS	4,713	2.01	0.956	−0.487	−34.947	0.000	SN
26.	MATH\AGREE\JOB INVOLVING MATHEMATICS	4,541	2.33	1.046	−0.173	−11.129	0.000	SN
27.	MATH\AGREE\NEED MAT TO GET THE JOB I WANT	4,569	1.70	0.879	−0.797	−61.280	0.000	SN
28.	MATH\AGREE\GOOD AT WORKING OUT PROBLEMS	4,588	2.26	0.946	−0.236	−16.880	0.000	SN
29.	MATH\AGREE\TEACHER EXPECTS TO DO	4,636	1.83	0.806	−0.670	−56.595	0.000	SN
30.	MATH\AGREE\PARENTS THINK MATH IMPORTANT	4,575	1.58	0.793	−0.923	−78.687	0.000	SN
31.	MATH\AGREE\USUALLY DO WELL IN MATH	4,642	1.85	0.826	−0.645	−53.267	0.000	SN

SP, significant positive; N, neutral; SN, significant negative. *p* < 0.05 confident, *p* > 0.05 not confident.

Factor 2: Students’ Safety and Behavior, a combination of 10 variables, i.e., BSBG16I, BSBG16G, BSBG16F, etc. The Factor 2 Cronbach’s alpha (α) value is 0.854, which is considered excellent and acceptable and is above 0.8, as reported by Bos and Kuiper (1999) and Cho (2011). For Factor 2: Students’ Safety and Behavior, each component variable loaded moderately high on the underlying factor (loadings between 0.322 and 0.738), indicating that they measure the underlying construct relatively well. The proportion of variation in Factor 2 explained by the component variables was 5.886%, which is moderate for a 10-variable scale. Cronbach’s alpha was 0.854, proving that the component variables are valid. These variables work well as a unit.

Factor 3: Attitude toward Math, a combination of seven variables, i.e., BSBM19HRSTUDENT, BSBM19CRSTUDENT, BSBM19ERSTUDENT, etc. The Factor 3 Cronbach’s alpha (α) value (attitude toward math) is 0.818, which is considered good and acceptable and is above 0.80, as reported by Cho (2010). For Factor 3: Attitude toward Math, each component variable loaded moderately high on the underlying factor (loadings between 0.322 and 0.738), demonstrating that they fairly accurately measure the underlying notion. A total of 4.897% of the variance in Factor 3 was explained by its constituent variables, which is relatively moderate for a 7-variable scale. Cronbach’s alpha was 0.818, proving that the component variables are valid. Component variables work well as a unit.

Factor 4: School and Classroom Environment, a combination of nine variables, i.e., BSBG15G, BSBG15F, BSBG15B, etc. The Factor 4 Cronbach’s alpha (α) value: school and classroom environment is 0.842, which is good and acceptable and demonstrates a high level of internal consistency among the factor’s variables. For Factor 4: School and Classroom Environment, each component variable loaded moderately on the underlying factor (loadings between 0.324 and 0.715), indicating that they measure the underlying construct relatively well. The percentage of Factor 4 variance that the component variables can account for was 3.792%, which is relatively low for a 9-variable scale. Cronbach’s alpha was 0.842, proving that the component variables are valid. Component variables work well as a unit.

Factor 5: Internet and Tablet A mix of 12 variables, such as BSBG06H, BSBG06G, BSBG06A, and so on. Cronbach’s alpha (α) value for Factor 5 Internet and Tablet for Math is 0.601, which is moderate and acceptable. For Factor 5: Internet and Tablet, each component variable loaded moderately on the underlying factor (loadings between 0.335 and 0.502), demonstrating that they fairly accurately measure the underlying notion. A total of 3.253% of the variance in Factor 5 was explained by its constituent variables, which is relatively low for a 12-variable scale, and Cronbach’s alpha was 0.601, providing further evidence that the component variables are valid. Component variables work well as a unit.

The results of the factor analysis show the internal consistency of Factors 1, 2, 3, and 4 is very high (0.95, 0.85, 0.82, and 0.84), and the items in the factor are closely related. The internal consistency of Factor 5 is moderate (0.60) compared to the internal consistency of Factors 1, 2, 3, and 4. The factor loading shows the variance explained by the variables on that particular factor. The loading factor of all items for Factor 1: Mathematics in School is high and acceptable. Also, the load factors of all items for Factor 2: Students’ Safety and Behavior, the load factors of all items for Factor 3: Attitude toward Math, the loading factor for Factor 4: School and Classroom Environment, and the loading factors of all items for Factor 5: Internet and Tablet are moderate and acceptable. The results showed Cronbach’s alpha (α) and loading factor were acceptable, and the related factor items are closely related.

Five new factors were created throughout the student questionnaire as a result of the factor analysis, which was entitled Factor 1: Mathematics in School, Factor 2: Students’ Safety and Behavior, Factor 3: Attitude toward Math, Factor 4: School and Classroom Environment, and Factor 5: Internet and Tablet. These factors were used in regression analysis to identify the most important student factors affecting student achievement on TIMSS 2015.

#### 3.1.1. One-sample *t*-test of student questionnaire: Factor 1 – Mathematics in School

A one-sample *t*-test was conducted to examine students’ perceptions of items related to Factor 1: Mathematics in School. These items had four-point Likert-scale responses ranging from strongly disagree (coded 4) to strongly agree (coded 1); the neutral value of 2.5 was used as a test value. The one-sample *t*-test showed that the rated items were lower than the neutral value. The highest-rated item was a favorite subject (mean = 2.37, SD = 1.131, and *p* < 0.05), and the lowest-rated item was that it was important to do well in math (mean = 1.57, SD = 0.794, and *p* < 0.05). Overall, the students had a negative attitude toward Factor 1: Mathematics in School (mean = 1.9552, SD = 0.62760, and *p* 0.05) (Table 5).

#### 3.1.2. One-sample *t*-test of the student questionnaire: Factor 2 – Safety and Behavior

A one-sample *t*-test was conducted to examine students’ perceptions of Factor 2: Safety and Behavior items. These items had 4-point Likert-scale responses, ranging from strongly disagree (coded 4) to strongly agree (coded 1).

The neutral value of 2.5 was used as the test value. The one-sample *t*-test shows that all the rated items were more than neutral. The highest-rated item was posted with embarrassing things (mean = 3.72, SD = 0.757, and *p* < 0.05), and the lowest-rated item was extra math lessons from the last 12 months (mean = 2.56, SD = 0.869, and *p* < 0.05). Overall, students had a positive perception toward Factor 2: Safety and Behavior (mean = 3.2490, SD = 0.65445, and *p* < 0.05) (Table 6).

**TABLE 6:** One-sample *t*-test and descriptive statistics for the components of Factor 2: safety and behavior.

No.	Items	*N*	Mean	SD	Mean difference	*t*-value	Significant (two-tailed)	Confident and not confident
**One-sample statistics and *t*-test (test value = 2.5)**								
1.	Factor 2: Safety and Behavior	4,781,	3.2490	0.65445	0.74900	79.134	0.000	SP
2.	GEN\HOW OFTEN\THREATENED	4,751,	3.60	0.858	1.095	87.969	0.000	SP
3.	GEN\HOW OFTEN\EMBARRASSING INFO	4,746,	3.45	0.964	0.949	67.829	0.000	SP
4.	GEN\HOW OFTEN\FORCE TO DO STH	4,752,	3.54	0.893	1.039	80.233	0.000	SP
5.	GEN\HOW OFTEN\HURT BY OTHERS	4,742,	3.35	1.005	0.851	58.307	0.000	SP
6.	GEN\HOW OFTEN\POSTED EMBARRASSING THINGS	4,756,	3.72	0.757	1.217	110.852	0.000	SP
7.	GEN\HOW OFTEN\SPREAD LIES ABOUT ME	4,694,	3.19	1.075	0.686	43.703	0.000	SP
8.	GEN\HOW OFTEN\LEFT OUT OF GAMES	4,724,	3.26	1.092	0.758	47.707	0.000	SP
9.	GEN\HOW OFTEN\MADE FUN OF	4,694,	2.75	1.231	0.248	13.809	0.000	SP
10.	GEN\HOW OFTEN\STOLE STH FROM ME	4,735,	3.24	1.061	0.744	48.245	0.000	SP
11.	MATH\EXTRA LESSONS LAST 12 MONTH\MATHEMATICS	4,530	2.56	0.869	0.362	28.079	0.000	SP

SP, significant positive; N, neutral; SN, significant negative. *p* < 0.05 confident, *p* > 0.05 not confident.

#### 3.1.3. One-sample *t*-test of student questionnaire: Factor 3 – Attitude toward Math

The perceptions of students toward items related to Factor 3: Attitude toward Math were investigated using a one-sample *t*-test. Responses to these questions ranged on a four-point Likert scale from strongly disagree (coded 4) to strongly agree (coded 1), with 2.5 as the test value. According to the one-sample *t*-test, all of the rated items were below neutral value. The item with the greatest rating was “math makes me confused” (mean = 2.35, SD = 1.666, and *p* 0.05), whereas the item with the lowest rating was “I wish I had not studied math” (mean = 2.18, SD = 1.089, and *p* 0.05). Students’ perceptions of Factor 3: Attitude toward Math were mostly negative (mean = 2.3458, SD = 0.69183, and *p* 0.05) (Table 7).

**TABLE 7:** One-sample *t*-test and descriptive statistics for the components of Student Factor 3 Attitude toward Math.

No.	Items	*N*	Mean	SD	Mean difference	*t*-value	Significant (tow-tailed)	Confident and not confident
**One-sample statistics and *t*-test (test value = 2.5)**								
1.	Factor 3: Attitude toward Math	4,758	2.3458	0.69183	−0.15424	−15.378	0.000	SN
2.	MATH\AGREE\MATHEMATICS IS HARDER FOR ME TO REVERSE	4,626	2.3240	1.05434	−0.17596	−11.351	0.000	SN
3.	MATH\AGREE\MATHEMATICS NOT MY STRENGTH REVERSE	4,574	2.2049	1.01304	−0.29515	−19.704	0.000	SN
4.	MATH\AGREE\MAT MAKES NERVOUS REVERSE	4,587	2.2906	1.01296	−0.20940	−14.000	0.000	SN
5.	MATH\AGREE\MATHEMATICS IS MORE DIFFICULT TO REVERSE	4,630	2.3395	0.99168	−0.16048	−11.011	0.000	SN
6.	MATH\AGREE\MATH IS BORING REVERSE	4,663	2.2644	1.02238	−0.23558	−15.735	0.000	SN
7.	MATH\AGREE\WISH HAVE NOT TO STUDY MATH REVERSE	4,716	2.1872	1.08939	−0.31277	−19.716	0.000	SN
8.	MATH\AGREE\MAHT MAKES CONFUSED REVERSE	4,613	2.357	1.66694	−0.33568	−34.185	0.000	SN

SP, significant positive; N, neutral, SN, significant negative. *p* < 0.05 confident, *p* > 0.05 not confident.

#### 3.1.4. One-sample *t*-test of the student questionnaire: Factor 4 – School and Classroom Environment

The students’ perceptions of the items relating to Factor 4: School and Classroom Environment were calculated using a one-sample test. Responses to these questions ranged on a four-point Likert scale from strongly disagree (coded 4) to strongly agree (coded 1), with 2.5 as the test value. The one-sample *t*-test reveals that the students substantially preferred one item on the arithmetic test that was in their native tongue (mean = 3.1992, SD = 0.98458, and *p* 0.05). However, students indicated negative opinions about eight criteria, including attending school (mean = 2.39, SD = 0.935, and *p* 0.05), seeing classmates (mean = 1.95, SD = 0.786, and *p* 0.05), and having fair professors. Overall, students’ opinions on Factor 4: School and Classroom Environment were unfavorable (mean = 2.4005, SD = 0.63389, and *p* 0.05) (Table 8).

**TABLE 8:** One-sample *t*-test for the components of Factor 4 and descriptive statistics: school and classroom environment.

No.	Items	*N*	Mean	SD	Mean difference	*t*-value	Significant (two-tailed)	Confident and not confident
**One-sample statistics and *t*-test (test value = 2.5)**								
1.	Factor 4: School and Classroom Environment	4781	20.4005	0.63389	−0.09949	−15.378	00.000	SN
2.	GEN\AGREE\LEARN A LOT	4,709	2.17	0.844	−0.326	−11.351	0.000	SN
3.	GEN\AGREE\PROUD TO GO TO THIS SCHOOL	4,700	2.31	0.952	−0.193	−19.704	0.000	SN
4.	GEN\AGREE\SAFE AT SCHOOL	4,698	2.33	0.927	−0.171	−14.000	0.000	SN
5.	GEN\AGREE\LIKE TO SEE CLASSMATES	4,688	1.95	0.786	−0.550	−11.011	0.000	SN
6.	GEN\AGREE\BELONG AT SCHOOL	4,648	2.41	0.934	−0.090	−15.735	0.000	SN
7.	GEN\AGREE\BEING IN SCHOOL	4,721	2.39	0.935	−0.108	−19.716	0.000	SN
8.	GEN\OFTEN SPEAK < LANG OF TEST > AT HOME REVERSE	4,745	3.1992	0.98458	0.69916	34.185	0.000	SP
9.	GEN\AGREE\FAIR TEACHERS	4,689	2.37	0.922	−0.125	−19.716	0.000	SN
10.	GEN\AGREE\LIKE TO SEE CLASSMATES	4,688	1.95	0.786	−0.550	−11.011	0.000	SN

SN, significant negative; N, neutral; SP, significant positive. *p* < 0.05 confident, *p* > 0.05 not confident.

#### 3.1.5. One-sample *t*-test of the student questionnaire: Factor 5 – Internet and Tablet

A one-sample *t*-test was conducted to examine students’ perceptions of Factor 5: the Internet and Tablet items. These items had a Likert scale, and the neutral value of 2.0 was used as the test value. The one-sample *t*-test shows that all the rated items had less than neutral values. The highest-rated item was how often to use a computer tablet (mean = 2.20, SD = 1.78, and *p* < 0.05), and the lowest-rated item was Internet connection (mean = 1.06, SD = 0.230, and *p* < 0.05). Overall, students had a negative perception toward Factor 5: Internet and Tablet (mean = 1.4664, SD = 0.24359, and *p* < 0.05). Factor 5: The Internet and Tablet align with the Deficit model in the conceptual framework of this study; for example, the shortage of the Internet, computers, tablets, and different school resources has a negative impact on students’ achievement (Table 9).

**TABLE 9:** One-sample *t*-test for the components of Factor 5 and descriptive statistics: Internet and tablet.

No.	Items	*N*	Mean	SD	Mean difference	*t*-value	Significant (two-tailed)	Confident and not confident
**One-sample statistics and *t*-test (test value = 2)**								
1.	Factor 5: Parents Beliefs	4,781	1.4664	0.24359	−0.53358	−151.460	0.000	SN
2.	GEN\HOME POSSESS\<COUNTRY SPECIFIC>	4,747	1.10	0.306	−0.395	−88.822	0.000	SN
3.	GEN\HOME POSSESS\GAMING SYSTEM	4,752	1.22	0.417	−0.276	−45.702	0.000	SN
4.	GEN\HOME POSSESS\COMPUTER TABLET OWN	4,753	1.15	0.355	−0.353	−68.531	0.000	SN
5.	GEN\HOME POSSESS\<COUNTRY SPECIFIC>	4,693	1.70	0.457	0.202	−30.272	0.000	SN
6.	GEN\HOME POSSESS\<COUNTRY SPECIFIC>	4,710	1.46	0.498	−0.042	−5.819	0.000	SN
7.	GEN\HOME POSSESS\OWN ROOM	4,726	1.42	0.493	−0.083	−11.594	0.000	SN
8.	GEN\HOME POSSESS\INTERNET CONNECTION	4,762	1.06	0.230	−0.444	−133.446	0.000	SN
9.	GEN\HOME POSSESS\OWN MOBILE PHONE	4,741	1.21	0.404	−0.294	−50.139	0.000	SN
10.	GEN\HOME POSSESS\<COUNTRY SPECIFIC>	4,738	1.70	0.457	0.202	−30.394	0.000	SN
11.	GEN\HOW OFTEN USE COMPUTER TABLET\OTHER	4,630	2.20	1.178	−0.303	−17.492	0.000	SN
12.	GEN\FATHER BORN IN\<COUNTRY>	4,741	1.70	0.644	−0.304	−32.523	0.000	SN
13.	GEN\MOTHER BORN IN\<COUNTRY>	4,751	1.70	0.633	−0.305	−33.190	0.000	SN

SN, significant negative; N, neutral; SP, significant positive. *p* < 0.05 confident, *p* > 0.05 not confident.

### 3.2. Student factors in multiple regression

The analysis in the current study involves multiple regression to investigate the influence of students, math teachers, and school factors on students’ achievement in TIMSS 2015. The TIMSS 2015 student performance was chosen as the dependent variable, and 15 variables were chosen as the independent variables. These were five aspects of the school, five aspects of the students, and five aspects of the math teachers. A viable analysis technique was determined to be multiple regression utilizing the entry approach (Darren and Paul, 2012). Prior to conducting the analysis, the pertinent statistical analysis assumptions were reviewed. In accordance with tests, the data did not exhibit multicollinearity (Coakes, 2009; Hair et al., 2011) and did not exhibit independent errors (Durbin-Watson = 1.527).

Further analysis of the standard residuals identified that the data obtained had no outliers (Std. Residual Min = −4.159, Std. Residual Max = 3.360). Scatter plots demonstrated that the assumptions of linearity and homogeneity were all satisfied (Hair et al., 2011). As all the assumptions remained encountered, the multiple regression analysis (*R*^2^) commenced; through a fixed order of entry, the extent to which the predictor variables predicted the criterion was determined (Table 10).

**TABLE 10:** One-way ANOVA.

Grades	Sum of squares	df	Mean square	*F*	Significant
Between groups	16,553.718	5	3,310.744	0.361	0.876
Within groups	266,456,192.100	29,023	9,180.863		
Total	266,472,745.900	29,028			

A one-way ANOVA was used to compare the differences between the average of the five probable values and the five plausible values. The first plausible value, the second plausible value, the third plausible value, the fourth plausible value, the fifth plausible value, and the average of the first five possible values were used to categorize student achievement into six categories. There were none of the anomalies in that order. Data were normally distributed for each group, as assessed by the Shapiro–Wilk test (*p* > 0.05), and variances were homogeneous, as assessed by Levene’s test of homogeneity of variances. In that order, a one-way ANOVA indicated that the differences between all five plausible values and the average of the five plausible value groups were not statistically significant.

To investigate the impact of student factors (Factor 1: Mathematics in School, Factor 2: Safety and Behavior, Factor 3: Attitude toward Math, Factor 4: School and Classroom Environment, and Factor 5: Internet and Tablet) on students’ achievement on TIMSS 2015, Five-stage Multiple Regression, the enter method was deemed a suitable method of analysis (Darren and Paul, 2012).

Multiple regression is used to clarify the dependent variable’s fluctuation by including additional independent variables. Multiple regression can also be used to calculate dependent variable values centered on updated values of the independent variables and to estimate the amount of change in the dependent variable when one unit of the independent variable changes. The proportion of the dependent variable is clarified in this unit, and new independent variables are included (Weisberg, 2014).

When explaining and stating findings from multiple regression, we recommend operating through three phases: (a) calculating the regression models that are meant for comparison; (b) deciding whether the multiple regression model is best for the information; and (c) comprehending the coefficients in the multiple regression model (Weisberg, 2014).

A separate five-stage multiple regression was conducted to investigate the effect of student factors on students’ achievement on TIMSS 2015. Factor 1: Mathematics in School was entered at stage one of the regressions as the main predictor to observe its effects on achievement in math in TIMSS 2015. Next, Factor 2: Safety and Behavior were entered at stage two. Next, Factor 3: Attitude toward Math was entered at stage three. Next, Factor 4: School and Classroom Environment, were entered at stage four, and Factor 5: Internet and Tablets, was entered at stage five. This order was deemed plausible for investigating the effects of student factors on students’ achievement in TIMSS 2015 Table 11.

**TABLE 11:** Multiple regression analysis between the five factors on student achievement in TIMSS 2015.

Model	*R*	*R* ^2^	Adjusted *R*	Standard error of the estimate	Change statistics					Durbin-Watson
					***R*^2^ change**	***F* change**	**df1**	**df2**	**Significant *F* change**	
1	0.271	0.074	0.073	89.04315	0.271	0.074	0.073	89.04315	0.271	
2	0.360	0.130	0.129	86.31248	0.360	0.130	0.129	86.31248	0.360	
3	0.425	0.181	0.180	83.73885	0.425	0.181	0.180	83.73885	0.425	
4	0.466	0.217	0.216	81.89306	0.466	0.217	0.216	81.89306	0.466	
5	0.478	0.228	0.228	81.29176	0.478	0.228	0.228	81.29176	0.478	1.288

a. Predictors: (constant), Factor 1: Mathematics in School.

b. Predictors: (constant), Factor 1: Mathematics in School, Factor 2: Safety and Behavior.

c. Predictors: (constant), Factor 1: Mathematics in School, Factor 2: Safety and Behavior, Factor 3: Attitude toward Math.

d. Predictors: (constant), Factor 1: Mathematics in School, Factor 2: Safety and Behavior, Factor 3: Attitude toward Math, Factor 4: School and Classroom Environment.

e. Predictors: (constant), Factor 1: Mathematics in School, Factor 2: Safety and Behavior, Factor 3: Attitude toward Math, Factor 4: School and Classroom Environment, Factor 5: Internet and Tablet.

f. Dependent variable: Achievement.

For step 1: *R* = 0.271, *R*^2^ = 0.074, Δ*R*^2^ = 0.271, *p* < 0.01; for step 2: *R* = 0.360, *R*^2^ = 0.130, Δ*R*^2^ = 0.360, *p* < 0.01; for step 3: *R* = 0.425, *R*^2^ = 0.181, Δ*R*^2^ = 0.425, *p* < 0.01; for step 4: *R* = 0.466, *R*^2^ = 0.217, Δ*R*^2^ = 0.466, *p* < 0.01; for step 5: *R* = 0.478, *R*^2^ = 0.228, Δ*R*^2^ = 0.478, *p* < 0.01.

The pertinent presumptions of this statistical analysis were examined before performing a multiple regression. To analyze five independent variables, a sample size of 4,751 was found sufficient. Green (1991) suggested 106 participants for this analysis, using the formula: *N* > 50 + 8m (where m is the number of independent variables). A statistically significant association between achievement and school-related characteristics was found after correlational analysis. However, the results of the collinearity tests supported the assumption that there was no multicollinearity (Coakes, 2005; Hair et al., 2011) [Factor 1: student factor F1 (mathematics in school), Tolerance = 0.848, VIF = 1.180; Factor 2: student factor F2 (safety and behavior), Tolerance = 0.937, VIF = 1.067; Factor 3: student factor F3 (Attitude toward Math), Tolerance = 0.861, VIF = 1.162; student Factor 4: mathematics helps students get a job]. The data also supported the independent error assumption (Durbin-Watson = 1.288). Analysis of the standard residuals revealed that there were no outliers in the data (Std. Residual Min = −3.836, Standard Residual Max = 3.486). The linearity assumptions were demonstrated via residual and scatter graphs, and homogeneity was all satisfied (Hair et al., 2011).

The multiple regression revealed that at Model 1, Factor 1: Mathematics in School contributed significantly to the regression model [*F*(1, 4,751) = 377.193, *p* < 0.01] the prediction of student’s achievement on TIMSS 2015 (Model 1) (*R*^2^ = 0.074) and accounted for approximately (7.4%) of the total variance in students’ achievement on TIMSS 2015 (Table 11). Adding Factor 2: Students’ Safety and Behavior to the prediction of achievement (Model 2) was an improvement over the earlier model, which led to a statistically significant increase in *R*^2^ of 0.130, *F* (2, 4,750) = 353.904, *p* < 0.01 since it could account for 13.0% of the total variance. The addition of Factor 3: Attitude toward Math to the prediction of achievement (Model 3) led to a statistically significant increase in *R*^2^ of 0.181, *F* (3, 4,749) = 349.814, *p* < 0.01, and accounted for 18.1% of the total variance. The addition of Factor 4: Students Mathematics help for Job to the prediction of achievement (Model 4) led to a statistically significant increase in *R*^2^ of 0.217, *F* (4, 4,748) = 328.693, *p* < 0.01 and accounted for 21.7% of the total Variance. The fifth and final model, comprised of five predictor factors (Factor 1: General School Resources, Factor 2: Discipline and Safety, Factor 3: Parental Support, Factor 4: Principal Experience and Education, and Factor 5: Internet and Tablet), with a prediction of students’ achievement on TIMSS 2015 (Model 5), led to a statistically significant increase in *R*^2^ of 0.228, *F* (5, 4,747) = 281.159, *p* < 0.01 and accounted for 22.8% of the total variance (Table 12).

**TABLE 12:** ANOVA results of the five student factors – model-multiple regression analysis.

Model		Sum of squares	df	Mean square	*F*	Significant
1	Regression	2,990,644.008	1	2,990,644.008	377.193	0.000
	Residual	37,669,171.960	4,751	7,928.683		
	Total	40,659,815.970	4,752			
2	Regression	5,273,054.696	2	2,636,527.348	353.904	0.000
	Residual	35,386,761.280	4,750	7,449.844		
	Total	40,659,815.970	4,752			
3	Regression	7,358,901.434	3	2,452,967.145	349.814	0.000
	Residual	33,300,914.540	4,749	7,012.195		
	Total	40,659,815.970	4,752			
4	Regression	8,817,484.044	4	2,204,371.011	328.693	0.000
	Residual	31,842,331.930	4,748	6,706.473		
	Total	40,659,815.970	4,752			
5	Regression	9,289,979.042	5	1,857,995.808	281.159	0.000
	Residual	31,369,836.930	4,747	6,608.350		
	Total	40,659,815.970	4,752	2,990,644.008	377.193	

a. Dependent variable: Achievement.

b. Predictors: (constant), Factor 1: Mathematics in School.

c. Predictors: (constant), Factor 1: Mathematics in School, Factor 2: Safety and Behavior.

d. Predictors: (constant), Factor 1: Mathematics in School, Factor 2: Safety and Behavior, Factor 3: Attitude toward Math.

e. Predictors: (constant), Factor 1: Mathematics in School, Factor 2: Safety and Behavior, Factor 3: Attitude toward Math, students F4 mathematics help students to get job.

f. Predictors: (constant), Factor 1: Mathematics in School, Factor 2: Safety and Behavior, Factor 3: Attitude toward Math, Factor 4: School and Classroom Environment, Factor 5: Internet and Tablet.

We learned the importance of each of the five models from the ANOVA result in Table 12 (one predictor, two predictors, three predictors, four predictors, and five predictors, respectively). All five models could be proven to be significant (*p* 0.01). Particularly, it was noted that model 1 with a single predictor had a higher *F* value. The *F* values, which were distinct from the *F* for the amount of change in achievement when adding a variable, were the overall predictive effects.

Models 1, 2, 3, 4, and 5’s *p*-values of 0.000 < 0.01 imply that the regression model is statistically significant. They indicate a significant linear relationship between achievement and mathematics in school, safety and behavior, attitude toward math, the school classroom environment, and the Internet and tablet (Table 13).

**TABLE 13:** Multiple regression analysis for five student predictor factors on student’s achievement on TIMSS 2015.

Model		Average of five plausible value		First plausible value		Second plausible value		Third plausible value		Fourth plausible value		Fifth plausible value	
		***B***	**Significant**	***B***	**Significant**	***B***	**Significant**	***B***	**Significant**	***B***	**Significant**	***B***	**Significant**
1	(Constant)	509.511	0.000	510.480	0.000	509.522	0.000	510.363	0.000	505.678	0.000	511.514	0.000
	F1: Mathematics in School	−40.010	0.000	−40.336	0.000	−40.027	0.000	−40.167	0.000	−38.875	0.000	−40.647	0.000
	*R* ^2^	0.074		0.070		0.067		0.067		0.063		0.071	
2	(Constant)	398.239	0.000	401.470	0.000	395.414	0.000	398.243	0.000	390.434	0.000	405.634	0.000
	F1: Mathematics in School	−38.888	0.000	−39.236	0.000	−38.876	0.000	−39.037	0.000	−37.713	0.000	−39.579	0.000
	F2: Safety and Behavior	33.577	0.000	32.894	0.000	34.433	0.000	33.833	0.000	34.775	0.000	31.950	0.000
	*R* ^2^	0.130		0.121		0.121		0.118		0.118		0.118	
3	(Constant)	482.797	0.000	486.775	0.000	479.373	0.000	482.178	0.000	475.208	0.000	490.450	0.000
	F1: Mathematics in School	−30.068	0.000	−30.338	0.000	−30.118	0.000	−30.281	0.000	−28.870	0.000	−30.731	0.000
	F2: Safety and Behavior	25.548	0.000	24.794	0.000	26.460	0.000	25.863	0.000	26.726	0.000	23.896	0.000
	F3: Attitude toward Math	−32.272	0.000	−32.557	0.000	−32.043	0.000	−32.034	0.000	−32.354	0.000	−32.370	0.000
	*R* ^2^	0.181		0.170		0.167		0.163		0.164		0.166	
4	(Constant)	543.551	0.000	546.841	0.000	539.001	0.000	543.882	0.000	538.297	0.000	549.734	0.000
	F1: Mathematics in School	−20.835	0.000	−21.209	0.000	−21.056	0.000	−20.904	0.000	−19.282	0.000	−21.722	0.000
	F2: Safety and Behavior	24.956	0.000	24.209	0.000	25.879	0.000	25.262	0.000	26.111	0.000	23.318	0.000
	F3: Attitude toward Math	−35.327	0.000	−35.577	0.000	−35.041	0.000	−35.137	0.000	−35.527	0.000	−35.351	0.000
	F4: School and Classroom Environment	−29.050	0.000	−28.721	0.000	−28.512	0.000	−29.505	0.000	−30.167	0.000	−28.347	0.000
	*R* ^2^	0.217		0.202		0.198		0.197		0.200		0.198	
5	(Constant)	478.081	0.000	485.258	0.000	471.245	0.000	477.718	0.000	469.730	0.000	486.454	0.000
	F1: Mathematics in School	−20.849	0.000	−21.223	0.000	−21.071	0.000	−20.918	0.000	−19.297	0.000	−21.736	0.000
	F2: Safety and Behavior	24.093	0.000	23.398	0.000	24.987	0.000	24.390	0.000	25.208	0.000	22.485	0.000
	F3: Attitude toward Math	−33.850	0.000	−34.189	0.000	−33.513	0.000	−33.645	0.000	−33.980	0.000	−33.924	0.000
	F4: School and Classroom Environment	−27.395	0.000	−27.164	0.000	−26.799	0.000	−27.832	0.000	−28.433	0.000	−26.748	0.000
	F5: Internet and Tablet	41.497	0.000	39.034	0.000	42.946	0.000	41.937	0.000	43.461	0.000	40.109	0.000
	*R* ^2^	0.228		0.212		0.210		0.207		0.211		0.208	

From Table 11, a one-way ANOVA showed no statistically significant difference between any of the five conceivable values and the average of the five probable value groups. According to the findings, the five TIMSS 2015 student achievement predictors and the constant had the following coefficients; Constant *B* = 478.081, *p* = 0.000: significant; Mathematics in School *B* = −20.849, *p* = 0.000: significant; Safety and Behavior *B* = 24.093, *p* = 0.000: significant; Attitude toward Math *B* = −33.850, *p* = 0.000: significant; School and Classroom Environment *B* = −27.395, *p* = 0.000: significant; Internet and Tablet *B* = 41.497, *p* = 0.000: significant.

The linear combination of the constant, the constant, and the TIMSS 2015 would be the model that fits the data best for forecasting kids’ performance on those tests. Factor 1: Mathematics in School, Factor 2: Safety and Behavior, Factor 3: Attitude toward Math, Factor 4: School and Classroom Environment, and Factor 5: The Internet and Tablets.

The coefficient estimate table for the multiple regression model is expressed as Achievement = 478.081–20.849 (Mathematics in School) + 24.093 (Safety and Behavior) – 33.850 (Attitude toward Math) − 27.395 (School and Classroom Environment) + 41.497 (Internet and Tablet).

This shows that achievement will drop by 20.849 for every unit improvement in students’ math proficiency at school. For every 1 unit increase in Safety and Behavior, the achievement will increase by 24.093. For every 1 unit increase in students’ Attitude toward Math, achievement will decline by 33.850. For every 1 unit increase in Students’ School and Classroom Environment, the achievement will decline by 27.395. Likewise, for every 1 unit increase in Internet and Tablets, the achievement will increase by 41.497.

Moreover, *p*-value = 0.000 < 0.01 for students’ Mathematics in School, Safety and Behavior, Attitude toward Math, School, and Classroom Environment, Internet and Tablet, respectively, implies that factors F1 (Mathematics in School) to F5 (Internet and Tablet) are statistically significant and therefore have a significant impact on achievement. Meanwhile, the variance inflation factor for Factor 1: Mathematics in School to Factor 5: Internet and Tablets are less than 5. There is no evidence of multicollinearity among the explanatory variables in this study, which supports the assumption that there should not be any. The histogram and P-P plot show that the data is approximately normally distributed, as there is no perfect normality in practice, which satisfies the normality assumptions. The partial regression plot shows that the scatter points all diffuse out, and no clear pattern satisfies the assumption of constant variance (homoscedasticity). Therefore, there is no evidence of heteroscedasticity.

## 4. Discussion

### 4.1. Mathematics in school

Mathematics in school significantly impacted students’ achievement in TIMSS 2015 [*B* = −22.817, *p* (0.000) < 0.01]. Furthermore, a one-sample *t*-test revealed that students negatively perceived Factor 1: Mathematics in School (mean = 1.9552, SD = 0.62760, and *p* 0.05).

This result is in line with the research by Goodall et al. (2017), who reported that many students in eighth grade usually do not have many opportunities to learn mathematics at home. Consequently, mathematics in school is essential because students can learn in a more organized manner. Teachers have already laid out syllabi to ensure students understand mathematics more effectively. Learning in school positively impacted students’ achievement on TIMSS 2015, mainly because they had qualified teachers to guide them.

Peer learning in eighth-grade mathematics also helps students learn with their team peers. Students who fail to understand any particular concept can obtain the necessary assistance from fellow students. This makes the school ideal for students to study (Eldeeb, 2012).

Similarly, Yaşaroğlu (2016) showed that school provides the necessary facilities for an eighth-grade student to learn effectively. Young students might require components such as counting aids, which are an integral part of mathematics learning (Afari, 2012) but are hardly available. Afari (2012) conducted a *Z*-test to determine whether counting aids assist eighth-grade students in performing better in mathematics. The results showed that the aids positively impacted the students’ understanding of mathematics, which was evident since they achieved an average mean of 2.78, which is >2.5. This result was confined to a school environment; in any other setting, the same result could not be achieved. Therefore, eighth-grade students learn mathematics more effectively when they learn in school, where all the components and amenities required for proper learning are readily available (Cuenca-Carlino et al., 2016).

It becomes evident from the preceding assertions about mathematics that the importance of mathematics in any society cannot be overemphasized. Von Suchodoletz et al. (2019) stated: “In today’s increasingly technological society, a strong background in mathematics is crucial for many career and job opportunities, like in the United Arab Emirates.” In his attempt to show how essential mathematics is, Abu-Hilal and Bahri (2000) asserted that mathematics is called the “queen of all sciences” since it has promoted the growth of many cultures. It is not only this but also the art of all arts. Wagie and Fox (2006) stated that math is considered the emperor of academia and the mirror of society. According to several schools of thought, mathematics is the foundation for all other subjects, including chemistry, physics, biology, and economics (Alharbi et al., 2020).

### 4.2. Student safety and behavior

In TIMSS 2015, students’ behavior and sense of safety had a substantial impact on their performance [*B* = −16.845, *p* (0.000) < 0.01]. Students’ perceptions of Factor 2: Safety and Behavior were good, according to a one-sample *t*-test (mean = 3.2490, SD = 0.65445, and *p* < 0.05).

This study showed that pride and safety are essential components of effective learning. Every student must feel confident and safe to comprehend what they are taught. When any student suffers from one or other forms of psychological or mental inferiority, it reflects in their learning because their pride has been dented, so their learning rate stagnates significantly. Pride, when accompanied by safety, enables harmony, which ultimately leads the student to perform at an optimal level, enabling achievement in the education process. This finding is consistent with Yaşaroğlu (2016), which shows that pride mainly results from students’ ability to solve academic issues they previously could not. The eighth-grade students are primarily concerned with what they can achieve. According to Cordero et al. (2018), solving a simple problem evokes a high sense of pride, which plays an essential role in the student’s achievement in TIMSS. For teachers and ordinary people not having any relationship with the education process, this could be regarded as insignificant, but for eighth-grade students, this achievement plays a significant role in boosting the student’s self-esteem (Khamis et al., 2008). In Abu Dhabi, the Emirate school’s pride and safety combination further encourages students to learn even more. A student who feels satisfied and safe will not mind working extra hard to understand different things. Consequently, every student in the eighth grade can benefit much more through pride and safety (Balfakih, 2010).

Achtziger and Gollwitzer (2018) found that, in addition to external threats such as school attacks and environmental dangers like natural disasters, secure learning environments can also be challenged by internal threats like bullying, physical punishment, and gang recruitment. All of these dangers have the potential to drastically lower students’ academic performance. According to Daleure et al. (2014), rising evidence links school environments and student results. However, much is still unclear about how perceived school safety affects learning. The majority of the research comes from middle- and high-income nations and is more concerned with educational outputs like attendance and retention than intellectual achievement (Ashour, 2020). Ridge (2009) discovered a more quantitative examination of the connection between school safety and student achievement in industrialized nations. Ridge (2009) concluded that students’ performance increases when they feel safe in school.

### 4.3. Attitude toward math

Attitude toward math is a crucial student factor that plays an integral role in determining students’ performance in eighth-grade mathematics. In TIMSS 2015, students’ attitudes toward mathematics had a big impact on their performance [*B* = −33.420, *p* (0.000) < 0.01]. Students’ attitudes toward Factor 3: Attitudes toward Math were perceived negatively, according to a one-sample *t*-test (mean = 2.3458, SD = 0.69183, and *p* 0.05).

Farooq and Shah (2008) concluded that there is a significant impact on a student’s confidence in mathematics. They used the Urdu-translated Fennema-Sherman Mathematics Attitude Scale to conduct a *t*-test with a *p* < 0.05. The results had an average mean of 1.276. They concluded that teachers and parents should focus mainly on boosting students’ confidence to improve their performance in mathematics. The study corresponds to a study by Di Martino and Zan (2011), which explains that a student’s emotions and beliefs impact their overall performance in mathematics. They concluded that students’ emotions and anxiety levels determine whether they will perform better in mathematics or not. Beliefs in mathematics are what students accept as being hard without even attempting a trial. To improve their performance, students should change their belief that mathematics is hard and complicated and instead develop the belief that it can be understood with practice and patience (Di Martino and Zan, 2011; Rodríguez and Sánchez, 2021).

Wardat et al. (2022a), stated that every student’s achievement in TIMSS is greatly affected by their attitude toward mathematics. The most critical period for homework is when the students have to be away from school for a prolonged period of time (Abdelfattah and Lam, 2018). This can occur over weekends or extended holiday breaks. Such times allow students to engage in activities that can easily erase what they have learned in school (Morgan, 2020).

Research by Hannula et al. (2014) indicated that the attitude toward mathematics students changes as they grow. Hannula explained that students’ progression from elementary to secondary school negatively impacts their mathematics learning. The study further explains that the general attitude toward mathematics is highly related to the quality of the socio-psychological climate and the teachings of the class (Hannula, 2002). They conducted a simple *t*-test to clarify the claim. The results were as follows: mean = 2.2615, SD = 0.6743, and *p* < 0.05. This shows that the socio-psychological climate negatively impacts students’ attitudes toward mathematics.

### 4.4. School and classroom environment

The results of the student questionnaire showed that students’ performance in TIMSS 2015 was strongly impacted by Factor 4: School and Classroom Environment (*B* = 5.743, *p* = 0.028 > 0.01). This finding is consistent with Goodall et al. (2017). Furthermore, a one-sample *t*-test reveals that students negatively perceived Factor 4: School and Classroom Environment (mean = 2.4005, SD = 0.63389, and *p* 0.05).

A student who understands this has a higher chance of working harder in mathematics to get a job after school (Eriksson et al., 2019). Teachers and other stakeholders have an essential role in ensuring they understand the importance of mathematics in getting jobs (Davis and Carlo, 2018). A student in the eighth grade might not fully comprehend this due to their immature nature. But according to the previous chapters of this dissertation, students who understand the job market have a greater opportunity to better understand mathematics. Some students might come from backgrounds where they do not need jobs to get the lives they want. Even such students should understand that jobs provide a unique opportunity to interact with other people, leading to happier lives (Gentilucci and Muto, 2007). Therefore, helping students understand that mathematics might enable them to advance their careers by making job opportunities readily available is among the most effective ways to help students learn more efficiently (Balfakih, 2010). Daleure et al. (2015) found that student achievement in mathematics is inextricably linked to future career opportunities, plays an essential role in the student’s general learning acquisitions, and is a reliable criterion to divide students into scientific or literary streams. Pauceanu et al. (2018) discovered that achievement in mathematics can open the door to well-compensated and prestigious career opportunities (Tashtoush et al., 2022a).

### 4.5. Internet and tablet

In TIMSS 2015, the Internet and tablets had a big influence on pupils’ academic performance [*B* = −33.420, *p* (0.000) < 0.01]. Students’ perceptions of Factor 5: Internet and Tablet use were negative, according to a one-sample *t*-test (mean = 1.4664, SD = 0.24359, and *p* 0.05). TIMSS 2015 showed that the Internet and tablets play an essential role in determining students’ performance. Burroughs et al. (2019) conducted a study to assess the effect of tablets on student achievement. They determined that tablet support improves students’ overall performance; the result showed a mean of 2.61, >2.5 (Burroughs et al., 2019).

This finding is consistent with that of Goodall’s (2017) study. Most technology resources support students’ achievement. According to Goodall (2017) and Dukmak and Ishtaiwa (2015), tablets and other technological resources have been found to encourage children to study mathematics and other subjects, leading to increased confidence among students. Dukmak and Ishtaiwa (2015) conducted a study to determine the impact of technological devices on students’ overall performance. The *Z*-test showed that technology devices supported mathematics in the students’ overall performance. Factor 5: In the conceptual framework of this study, the Internet and tablets are consistent with the Deficit Model; for example, the shortage of the Internet, computers, tablets, and different school resources has a negative impact on students’ achievement.

## 5. Conclusion

Trends in International Mathematics and Science Study 2015 results were focused on students, mathematics teachers, and school questionnaires and their effects on student achievement.

A PCA was run on a 90-item questionnaire that asked students in Abu Dhabi public and private schools to provide information about aspects of their home and school lives, including home environment, school climate for learning, self-perception, and attitudes toward learning mathematics. The PCA revealed the following five factors: (Factor 1: Mathematics in School, Factor 2: Students’ Safety and Behavior, Factor 3: Attitude toward Mathematics, Factor 4: School and Classroom Environment, and Factor 5: Internet and Tablet Usage).

A one-sample *t*-test was calculated to examine the perceptions of students on items related to Factor 1, namely: Mathematics in School, Factor 2: Safety and Behavior, Factor 3: Attitude toward Mathematics, Factor 4: School and Classroom Environment, and Factor 5: Internet and Tablets usage and effects on students’ achievement in TIMSS 2015.

The one-sample *t*-test revealed that students had a negative perception toward Factor 1: Mathematics in School (mean = 1.9552, SD = 0.62760, and *p* < 0.05), students had a positive perception toward Factor 2: Safety and Behavior (mean = 3.2490, SD = 0.65445, and *p* < 0.05), students had a negative perception toward Factor 3: Attitude toward Mathematics (mean = 2.3458, SD = 0.69183, and *p* < 0.05), students had a negative perception toward Factor 4: School and Classroom Environment (mean = 2.4005, SD = 0.63389, and *p* < 0.05), and students had a negative perception toward Factor 5: Internet and Tablet (mean = 1.4664, SD = 0.24359, and *p* < 0.05).

To investigate the effects of students’ factors (Factor 1: Mathematics in School, Factor 2: Safety and Behavior, Factor 3: Attitude toward Mathematics, Factor 4: School and Classroom Environment, and Factor 5: Internet and Tablet) on students’ achievement in TIMSS 2015, a five-stage multiple regression using the enter method was deemed a suitable method of analysis (Darren and Paul, 2012).

The full model of student factors’ multiple regression revealed that all the student factors were statistically significant predictors of student achievement in TIMSS 2015. This implies that Mathematics in School, Safety and Behavior, Attitude toward Mathematics, School, Classroom Environment, and Internet and Tablet usage significantly impacted students’ achievement in TIMSS 2015 (Tashtoush et al., 2022b).

## Data availability statement

The data for the study is publicly available from TIMSS & PIRLS International Study Center, Lynch School of Education, Boston College (https://timssandpirls.bc.edu/timss2015/international-database/).

## Ethics statement

The study was conducted in accordance with the Declaration of Helsinki, and approved by the Ethics Committee of United Arab Emirates University protocol code ERS_2020_6205 and date of approval was 22 October 2020. Written informed consent was not required for this study in accordance with the national legislation and the institutional requirements.

## Author contributions

YW and HT: conceptualization. YW, HT, and HD: methodology. YW and SB: software, formal analysis, data curation, and project administration. YW, SB, HT, RT, ME, and HD: validation. YW: investigation and writing—original draft preparation. YW, SB, HT, RT, and ME: resources. SB, HT, RT, ME, and HD: writing—review and editing, and supervision. YW, SB, and HD: visualization and funding acquisition. All authors had read and agreed to the published version of the manuscript.

## Footnotes

1 Timssandpirls.bc.edu, 2015.

## References

[B1] Abdelfattah F. Lam J. (2018). Linking homework to achievement in mathematics: an examination of 8th-grade Arab participation in TIMSS 2015. *Int. J. Instruction* 11 607–624. 10.12973/iji.2018.11438a

[B2] Abu-Hilal M. M. Bahri T. M. (2000). Self-concept: the generalizability of research on the SDQ, marsh/shavelson model, and I/E frame of reference model to United Arab emirates students. *Soc. Behav. Pers.* 28 309–322. 10.2224/sbp.2000.28.4.309

[B3] Acharya B. R. (2017). Factors affecting difficulties in learning mathematics by mathematics learners. *Int. J. Elemen. Educ*. 6, 8–15. 10.11648/j.ijeedu.20170602.11

[B4] Achtziger A. Gollwitzer P. M. (2018). *Motivation and volition in the course of action*. Cham: Springer International Publishing, 485–527.

[B5] Achtziger A. Gollwitzer R. M. (2008). “Motivation and volition in the course of action,” in *Motivation and Action*, eds Heckhausen J. Heckhausen H. (Cambridge: Cambridge University Press), 272–295. 10.1017/CBO9780511499821.012

[B6] Afari E. (2012). Examining the factorial validity of the attitudes towards mathematics inventory (ATMI) in the United Arab emirates: a confirmatory factor analysis. *Int. Rev. Contemp. Learn. Res.* 2 15–29. 10.12785/irclr/020102

[B7] Allison P. D. (2001). *Missing data*. Thousand Oaks, CA: Sage Publications.

[B8] Al Shannag Q. A. Tairab H. Dodeen H. Abdel-Fattah F. (2013). Linking teachers’ quality and student achievement in the Kingdom of Saudi Arabia and Singapore: the impact of teachers’ background variables on student achievement. *J. Baltic Sci. Educ.* 12:652. 10.33225/jbse/13.12.652

[B9] Alharbi M. S. Almatham K. A. Alsalouli M. S. Hussein H. B. (2020). Mathematics teachers’ professional traits that affect mathematical achievement for fourth-grade students according to the TIMSS 2015 Results: a comparative study among Singapore, Hong Kong, Japan, and Saudi Arabia. *Int. J. Educ. Res.* 104 1–15. 10.1016/j.ijer.2020.101671

[B10] Ashour S. (2020). Analysis of the attrition phenomenon through the lens of university dropouts in the United Arab emirates. *J. Appl. Res. High. Educ.* 12 357–374. 10.1108/JARHE-05-2019-0110

[B11] Balfakih N. M. A. (2010). The effectiveness of student team-achievement division (STAD) for teaching high school chemistry in the United Arab Emirates. *Int. J. Sci. Educ.* 25 605–624. 10.1080/09500690110078879

[B12] Bos K. Kuiper W. (1999). Modelling TIMSS data in a European comparative perspective: exploring influencing factors on achievement in mathematics in grade 8. *Educ. Res. Eval.* 5 157–179. 10.1076/edre.5.2.157.6946

[B13] Bryk A. S. Raudenbush S. W. (1988). Toward a more appropriate conceptualization of research on school effects: A three-level hierarchical linear model. *Am. J. Educ.* 97, 65–108. 10.1086/443913

[B14] Burroughs N. Gardner J. Lee Y. Guo S. Touitou I. Jansen K. (2019). “A review of the literature on teacher effectiveness and student outcomes,” in *Teaching for excellence and equity. IEA research for education*, Vol. 6, (Berlin: Springer Nature), 7–17. 10.1007/978-3-030-16151-4_2

[B15] Cattell R. B. (1966). The scree test for the number of factors. *Multivariate Behav. Res.* 1 245–276. 10.1207/s15327906mbr0102_10 26828106

[B100] Cho C. (2010). Korean wave in Malaysia and changes of the Korean-Malaysia relations. *Malaysian J. Media Stud*. 12, 1–14.

[B16] Cho M. O. (2011). *A Comparison of the Effectiveness of Science Education in Korea and South Africa: a Multilevel Analysis of TIMSS 2003 Data.* Doctoral dissertation. Pretoria: University of Pretoria.

[B17] Coakes S. J. (2005). *SPSS. Singapore*. Melbourne, VIC: John Wiley and Sons Australis, Ltd.

[B18] Coakes S. J. (2009). *SPSS: Analysis Without Anguish : Version 16 for Windows.* Milton: John Wiley & Sons Australia.

[B19] Cordero J. M. Cristobal V. Santín D. (2018). Causal inference on education policies: a survey of empirical studies using PISA, TIMSS and PIRLS. *J. Econ. Surveys* 32 878–915. 10.1111/joes.12217

[B20] Cuenca-Carlino Y. Freeman-Green S. Stephenson G. W. Hauth C. (2016). Self-regulated strategy development instruction for teaching multi-step equations to middle school students struggling in math. *J. Special Educ.* 50 75–85. 10.1177/0022466915622021

[B21] Daleure G. Albon R. Hinkston K. McKeown J. Zaabi T. A. (2015). *Understanding family involvement in the education of Emirati college students in the United Arab Emirates (UAE). Intercultural communication with Arabs: Studies in educational, professional and societal contexts*, 77–108.

[B22] Daleure G. M. Albon R. Hinkston K. Ajaif T. McKeown J. (2014). Family involvement in emirati college student education and linkages to high and low achievement in the context of the United Arab emirates. *FIRE Forum Int. Res. Educ.* 1 8–31. 10.18275/fire201401031024

[B23] Darren G. Paul M. (2012). *IBM SPSS Statistics 19 Step by Step. a Simple Guide and Reference*, 12th Edn. London: Pearson.

[B24] Davis A. N. Carlo G. (2018). The roles of parenting practices, sociocognitive/emotive traits, and prosocial behaviors in low-income adolescents. *J. Adoles.* 62 140–150. 10.1016/j.adolescence.2017.11.011 29197236

[B25] Di Martino P. Zan R. (2011). Attitude towards mathematics: a bridge between beliefs and emotions. *ZDM Math. Educ.* 43 471–482. 10.1007/s11858-011-0309-6

[B26] Dukmak S. Ishtaiwa F. F. (2015). Factors influencing the academic achievement of students in the preparatory and secondary schools of the United Arab emirates. *Eur. J. Soc. Sci.* 46 132–148.

[B27] Eldeeb A. M. Z. (2012). *The Impact of Parental Involvement on Academic Student Achievement.* doctoral dissertation. Dubai: The British University in Dubai.

[B28] Eriksson K. Helenius O. Ryve A. (2019). Using TIMSS items to evaluate the effectiveness of different instructional practices. *Instr. Sci.* 47 1–18. 10.1007/s11251-018-9473-1

[B29] Farooq M. S. Chaudhry A. H. Shafiq M. Berhanu G. (2011). Factors affecting students’ quality of academic performance: a case of secondary school level. *J. Qual. Technol. Manag.* 7 1–14. 27885969

[B30] Farooq M. S. Shah S. Z. U. (2008). Students ‘attitude towards mathematics. *Pak. Econ. Soc. Rev.* 46, 75–83.

[B31] Fulmer G. W. Tanas J. Weiss K. A. (2018). The challenges of alignment for the next generation science standards. *J. Res. Sci. Teaching* 55 1076–1100. 10.1002/tea.21481

[B32] Fung F. Tan C. Y. Chen G. (2018). Student engagement and mathematics achievement: unraveling main and interactive effects. *Psychol. Schools* 55 815–831. 10.1002/pits.22139

[B33] Gentilucci J. L. Muto C. C. (2007). Principals’ influence on academic achievement: the student perspective. *NASSP Bull.* 91 219–236. 10.1177/0192636507303738

[B34] Goodall C. (2018). Inclusion is a feeling, not a place: a qualitative study exploring autistic young people’s conceptualisations of inclusion. *Int. J. Inclusive Educ.* 24 1285–1310. 10.1080/13603116.2018.1523475

[B35] Goodall J. (2017). *Narrowing the Achievement Gap: Parental Engagement with Children’s Learning.* Abingdon: Routledge. 10.4324/9781315672465

[B36] Goodall S. Thomas K. Harper L. D. Hunter R. Parker P. Stevenson E. (2017). The assessment of neuromuscular fatigue during 120 min of simulated soccer exercise. *Eur. J. Appl. Physiol*. 117, 687–697. 10.1007/s00421-017-3561-9 28247027

[B37] Green S. B. (1991). How many subjects does it take to do a regression analysis. *Multivar. Behav. Res*. 26, 499–510. 10.1207/s15327906mbr2603_7 26776715

[B38] Hannula M. S. (2002). Attitude towards mathematics: emotions, expectations and values. *Educ. Stud. Math.* 49 25–46. 10.1023/A:1016048823497

[B39] Hannula M. S. Bofah E. Tuohilampi L. Metsämuuronen J. (2014). “A longitudinal analysis of the relationship between mathematics-related affect and achievement in finland,” in *Proceedings of the 27th Conference of the International Group for the Psychology of Mathematics Education, Vancouver*, eds Oesterle S. Liljedahl P. Nicol C. Allan D. (Canada: PME).

[B40] Hair J. Ringle C. Sarstedt M. (2011). PLS-SEM: Indeed a silver bullet. *J. Mark. Theory Pract*. 19, 139–151. 10.2753/MTP1069-6679190202

[B41] Harris D. N. Sass T. R. (2011). Teacher training, teacher quality and student achievement. *J. Public Econ.* 95 798–812. 10.1016/j.jpubeco.2010.11.009

[B42] Herges R. M. Duffied S. Martin W. Wageman J. J. (2017). Motivation and achievement of middle school mathematics students. *Math. Educator* 26 83–106.

[B43] Johansson S. (2016). International large-scale assessments: What uses, what consequences? *Educ. Res*. 58, 139–148.

[B44] Kaiser H. F. (1960). The application of electronic computers to factor analysis. *Educ. Psychol. Meas.* 20 141–151. 10.1177/001316446002000116

[B45] Khamis V. Dukmak S. Elhoweris H. (2008). Factors affecting the motivation to learn among United Arab Emirates middle and high school students. *Educ. Stud*. 34, 191–200.

[B46] Kromrey J. D. Rendina-Gobioff G. (2006). On knowing what we do not know: An empirical comparison of methods to detect publication bias in meta-analysis. *Educ. Psychol. Measur*. 66, 357–373.

[B47] Lamb S. Fullarton S. (2002). Classroom and school factors affecting mathematics achievement: a comparative study of Australia and the United States using TIMSS. *Aust. J. Educ.* 46 154–171. 10.1177/000494410204600205

[B48] Lee J. Stankov L. (2018). Non-cognitive predictors of academic achievement: Evidence from TIMSS and PISA. *Learn. Individ. Differ*. 65, 50–64. 10.1016/j.lindif.2018.05.009

[B49] Martin M. O. Mullis I. V. Foy P. Stanco G. M. (2012). *TIMSS 2011 international results in science. International association for the evaluation of educational achievement*. Available online at: https://timssandpirls.bc.edu/timss2011/downloads/T11_IR_Science_FullBook.pdf

[B50] Martin M. O. Mullis I. V. S. Hooper M. (Eds). (2016). *Methods and procedures in TIMSS 2015. Retrieved from Boston college, TIMSS & PIRLS international study center*. Available online at: http://timssandpirls.bc.edu/publications/timss/2015-methods.html

[B51] Morgan H. (2020). Best practices for implementing remote learning during a pandemic. *Clearing House J. Educ. Strategies Issues Ideas* 93 135–141. 10.1080/00098655.2020.1751480

[B52] Mullis I. V. Martin M. O. (2012). Using TIMSS and PIRLS to improve teaching and learning. *Recherches en Éduc.* 10.4000/ree.5835

[B53] Mullis I. V. Martin M. O. Foy P. Hooper M. (2016). *TIMSS advanced 2015 International Results in Advanced Mathematics and Physics. TIMSS and PIRLS International Study Centere, Lynch School of Education, Boston College.* Available online at: http://timssandpirls.bc.edu/timss2015/international-results/wp-content/uploads/filebase/advanced/full%20pdfs/TA15-International-Results-in-Advanced-Mathematics-and-Physics.pdf

[B54] National Center for Education Statistics (2020). *Trends in International Mathematics and Science Study (TIMSS): Participating Countries.* Available online at: https://nces.ed.gov/timss/participation.asp

[B55] Pauceanu A. Alpenidze O. Edu T. Zaharia R. (2018). What determinants influence students to start their own business? empirical evidence from United Arab Emirates universities. *Sustainability* 11:92. 10.3390/su11010092

[B56] Ridge T. (2009). *Living with Poverty: a Review of the Literature on Children’s and Families’ Experiences of Poverty. Research Report No 594.* Norwich: Department for Work and Pensions, HMSO.

[B57] Rodríguez M. Sánchez R. (2021). *Attitudes and Preferences Towards Mathematics in Students and its Incidence in the Teaching-Learning Process, Hipótese Magazine, Edition.* Available online at: https://revistahipotese.webnode.com/edicao-2021/

[B58] Straub D. Boudreau M. Gefen D. (2004). Validation guidelines for is positivist research. *Commun. Associat. Inform. Syst*. 13, 380–427.

[B59] Tashtoush M. A. Wardat Y. Aloufi F. Taani O. (2022a). The effectiveness of teaching method based on the components of concept-rich instruction approach in students achievement on linear algebra course and their attitudes towards mathematics. *J. High. Educ. Theory Pract.* 22 41–57. 10.33423/jhetp.v22i7.5269

[B60] Tashtoush M. A. Wardat Y. Aloufi F. Taani O. (2022b). The effect of a training program based on TIMSS to developing the levels of habits of mind and mathematical reasoning skills among pre-service mathematics teachers. *Eurasia J. Math. Sci. Technol. Educ.* 18:em2182. 10.29333/ejmste/12557

[B61] Thurstone L. L. (1947). *Multiple Factor Analysis: a Development and Expansion of Vectors of the Mind.* Chicago: University of Chicago Press.

[B62] Von Suchodoletz A. Rahn J. Nadyukova I. Barza L. Achtziger A. (2019). Can mindsets influence college students’ motivation to learn? findings from the United States and the United Arab emirates. *High. Educ.* 79 731–748. 10.1007/s10734-019-00434-z

[B63] Wagie D. Fox W. (2006). Transforming higher education in the United Arab emirates (UAE): contributing to social progress and the new economy. *Int. J. Learn. Annu. Rev.* 12 277–286. 10.18848/1447-9494/CGP/v12i07/47926

[B64] Wardat Y. Belbase S. Tairab H. Takriti R. A. Efstratopoulou M. Dodeen H. (2022b). The influence of school factors on students’ mathematics achievements in trends in international mathematics and science study (TIMSS) in Abu Dhabi Emirate Schools. *Educ. Sci.* 12:424. 10.3390/educsci12070424 PMC10206125 37235093

[B65] Wardat Y. Belbase S. Tairab H. (2022a). Mathematics teachers’ perceptions of trends in international mathematics and science study (TIMSS)-related practices in Abu Dhabi emirateschools. *Sustainability* 14:5436. 10.3390/su14095436

[B66] Weisberg S. (2014). *Applied Linear Regression.* Hoboken, NJ: John Wiley & Sons.

[B67] Yalcin S. Demirtasli R. N. Dibek M. I. Yavuz H. C. (2017). The effect of teacher and student characteristics on TIMSS 2011 mathematics achievement of fourth-and eighth-grade students in Turkey. *Int. J. Progressive Educ.* 13 79–94.

[B68] Yaşaroğlu C. (2016). Cooperation and importance of school and family on values education. *Eur. J. Multidiscip. Stud.* 1 66–71. 10.26417/ejms.v1i2.p66-71

